# {2-[(2-Acetyl­hydrazin-1-yl­idene)methyl-κ^2^ *N* ^1^,*O*]-6-methoxy­phenolato-κ*O* ^1^}(nitrato-κ*O*)copper(II) monohydrate

**DOI:** 10.1107/S1600536810000632

**Published:** 2010-01-09

**Authors:** Ibrahima Elhadj Thiam, Pascal Retailleau, Alda Navaza, Mohamed Gaye

**Affiliations:** a Département de Chimie, Faculté des Sciences et Techniques, Université Cheikh Anta Diop, Dakar, Senegal; b ICSN-CNRS, Laboratoire de Cristallochimie, 1 Avenue de la Terasse, 91198 Gif-sur-Yvette, France; c ANBioPhi FRE 3207 CNRS, Université de Paris 13, 74 Rue Marcel Cachin, 93017 Bobigny, France

## Abstract

In the title complex, [Cu(C_10_H_11_N_2_O_3_)(NO_3_)]·H_2_O, prepared from the Schiff base *N*′-(3-meth­oxy-2-oxidobenzyl­idene)­acetohydrazide, the Cu^II^ atom is coordinated by two O atoms and one N atom from the ligand and one O atom from a nitrate group in a distorted square-planar geometry. The Cu^II^ atom has a weak inter­action with another O atom of the nitrate group. The two O atoms of the tridentate Schiff base ligand are in a *trans* arrangement. O—H⋯O and N—H⋯O hydrogen bonds involving the uncoordinated water mol­ecule are observed.

## Related literature

For related structures, see: Ainscough *et al.* (1998 ▶); Koh *et al.* (1998 ▶); Tamboura *et al.* (2009 ▶); You & Zhu (2004 ▶).
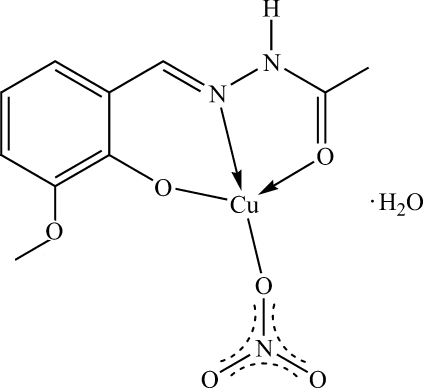


## Experimental

### 

#### Crystal data

[Cu(C_10_H_11_N_2_O_3_)(NO_3_)]·H_2_O
*M* *_r_* = 350.77
Monoclinic, 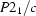

*a* = 9.274 (2) Å
*b* = 10.455 (4) Å
*c* = 13.726 (4) Å
β = 95.16 (5)°
*V* = 1325.5 (7) Å^3^
*Z* = 4
Mo *K*α radiation
μ = 1.69 mm^−1^
*T* = 293 K
0.40 × 0.28 × 0.20 mm

#### Data collection

Nonius KappaCCD diffractometer
Absorption correction: multi-scan (*DENZO*/*SCALEPACK*; Otwinowski & Minor, 1997 ▶) *T*_min_ = 0.56, *T*_max_ = 0.72
5500 measured reflections
3046 independent reflections
2493 reflections with *I* > 2σ(*I*)
*R*_int_ = 0.021

#### Refinement

*R*[*F* ^2^ > 2σ(*F* ^2^)] = 0.033
*wR*(*F* ^2^) = 0.090
*S* = 1.05
3046 reflections
192 parameters
H-atom parameters constrained
Δρ_max_ = 0.33 e Å^−3^
Δρ_min_ = −0.44 e Å^−3^

### 

Data collection: *COLLECT* (Nonius, 1998 ▶); cell refinement: *DENZO*/*SCALEPACK* (Otwinowski & Minor, 1997 ▶); data reduction: *DENZO*/*SCALEPACK*; program(s) used to solve structure: *SHELXS97* (Sheldrick, 2008 ▶); program(s) used to refine structure: *SHELXL97* (Sheldrick, 2008 ▶); molecular graphics: *PLATON* (Spek, 2009 ▶); software used to prepare material for publication: *SHELXL97*.

## Supplementary Material

Crystal structure: contains datablocks I, global. DOI: 10.1107/S1600536810000632/hy2267sup1.cif

Structure factors: contains datablocks I. DOI: 10.1107/S1600536810000632/hy2267Isup2.hkl

Additional supplementary materials:  crystallographic information; 3D view; checkCIF report

## Figures and Tables

**Table 1:** Selected bond lengths (Å)

Cu1—N1	1.9134 (18)
Cu1—O1	1.8798 (15)
Cu1—O3	1.9730 (16)
Cu1—O4	1.9663 (16)
Cu1—O6	2.559 (2)

**Table 2:** Hydrogen-bond geometry (Å, °)

*D*—H⋯*A*	*D*—H	H⋯*A*	*D*⋯*A*	*D*—H⋯*A*
N2—H2N⋯O7	0.86	1.95	2.801 (3)	174
O7—H1O⋯O1^i^	0.92	2.40	3.271 (3)	159
O7—H1O⋯O2^i^	0.92	2.42	3.050 (3)	126
O7—H2O⋯O5^ii^	0.92	2.08	2.984 (3)	167

Symmetry codes: (i) 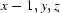; (ii) 
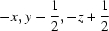
.

**Fig. 1.**
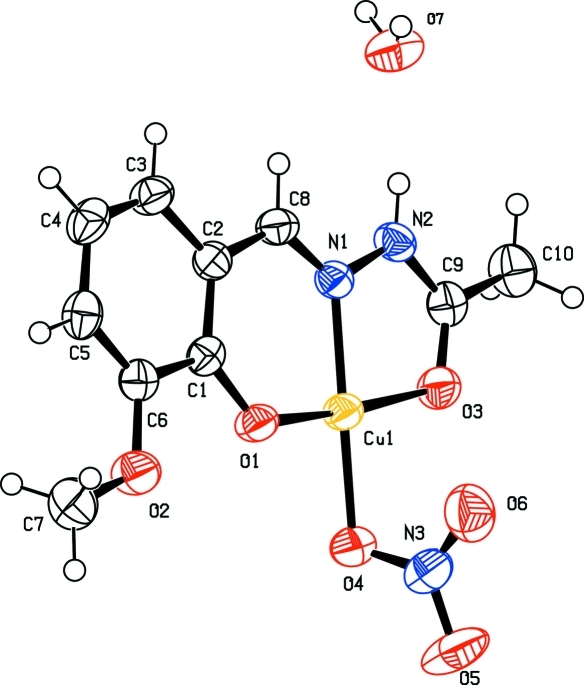
Molecular structure of the title compound. Displacement ellipsoids are drawn at the 50% probability level.

## supplementary crystallographic information

### Comment

In the title complex, the Cu^II^ ion adopts a four-coordinated geometry with the Schiff base coordinated to the metal ion as a uninegative charged tridentate ligand via the carbonyl O atom, the azomethine N atom and the phenolate O atom. The fourth coordination position is occupied by an O atom of the nitrate group. The Cu^II^ ion has a weak interaction with another O atom (O6) of the nitrate (Table 1). The bond distances of Cu—N and Cu—O are similar to the other Cu analogue with the same tridentate ligand (Ainscough *et al.*, 1998). The Cu—O(NO_3_) distance is similar to the observed value for the complex [Cu(*L*)NO_3_] [*L* = 1-(pyridin-2-ylmethyliminomethyl)naphtalen-2-olato] (You & Zhu, 2004). The two O donor atoms of the ligand are in a *trans* arrangement with an O—Cu—O angle of 173.76 (6)°. The angles around Cu are in a range of 81.49 (7)–173.76 (6)° and sum of the angles at Cu is 360.4°, suggesting that the geometry around the Cu atom is distorted square-planar (Fig. 1).

### Experimental

All purchased chemicals and solvents were reagent grade and used without further purification. The solid-state IR spectra were recorded from KBr discs on a Nicolet spectrophotometer. To a mixture of the ligand (0.211 g, 1.0 mmol) and 20 ml of ethanol was added dropwise a solution of copper nitrate dihydrate (0.242 g, 2.0 mmol) in 10 ml of ethanol. The resulting mixture was stirred under reflux for 2 h. After cooling the solution was filtered and the filtrate was left for slow evaporation. Green crystals of the title compound were obtained in good yield (0.290 g, 82.7%). IR (cm^-1^): 3403, 1604, 1578, 1445, 1291, 1248, 1082, 1004, 331, 273. Melting point 196±1°C. Analysis, calculated for C_10_H_13_CuN_3_O_7_: C 34.24, H 3.74, N 11.98%; found: C 34.26, H 3.73, N 16.15%. Single crystals suitable for X-ray analysis were obtained from slow evaporation of a methanol solution of the product.

### Refinement

Water H atoms and amine H atoms of the Schiff base ligand were located from a difference Fourier map and refined as riding atoms with *U*_iso_(H) = 1.2*U*_eq_(N,O). Other H atoms were placed geometrically and refined with a riding model, with C—H = 0.93 (CH) and 0.96 (CH_3_) Å and with *U*_iso_(H) = 1.2(1.5 for methyl)*U*_eq_(C).

### Crystal data

**Table:**

[Cu(C_10_H_11_N_2_O_3_)(NO_3_)]·H_2_O	*F*(000) = 716
*M**_r_* = 350.77	*D*_x_ = 1.758 Mg m^−^^3^
Monoclinic, *P*2_1_/*c*	Melting point: 469 K
Hall symbol: -P 2ybc	Mo *K*α radiation, λ = 0.71070 Å
*a* = 9.274 (2) Å	Cell parameters from 12725 reflections
*b* = 10.455 (4) Å	θ = 1.0–27.5°
*c* = 13.726 (4) Å	µ = 1.69 mm^−^^1^
β = 95.16 (5)°	*T* = 293 K
*V* = 1325.5 (7) Å^3^	Prism, green
*Z* = 4	0.40 × 0.28 × 0.20 mm

### Data collection

**Table:**

Nonius KappaCCD diffractometer	3046 independent reflections
Radiation source: fine-focus sealed tube	2493 reflections with *I* > 2σ(*I*)
graphite	*R*_int_ = 0.021
φ and ω scans	θ_max_ = 27.5°, θ_min_ = 2.5°
Absorption correction: multi-scan (*DENZO*/*SCALEPACK*; Otwinowski & Minor, 1997)	*h* = −12→12
*T*_min_ = 0.56, *T*_max_ = 0.72	*k* = −13→12
5500 measured reflections	*l* = −17→17

### Refinement

**Table:**

Refinement on *F*^2^	Primary atom site location: structure-invariant direct methods
Least-squares matrix: full	Secondary atom site location: difference Fourier map
*R*[*F*^2^ > 2σ(*F*^2^)] = 0.033	Hydrogen site location: inferred from neighbouring sites
*wR*(*F*^2^) = 0.090	H-atom parameters constrained
*S* = 1.05	*w* = 1/[σ^2^(*F*_o_^2^) + (0.0515*P*)^2^ + 0.2512*P*] where *P* = (*F*_o_^2^ + 2*F*_c_^2^)/3
3046 reflections	(Δ/σ)_max_ = 0.001
192 parameters	Δρ_max_ = 0.33 e Å^−^^3^
0 restraints	Δρ_min_ = −0.44 e Å^−^^3^

### Fractional atomic coordinates and isotropic or equivalent isotropic displacement parameters (Å^2^)

**Table:**

	*x*	*y*	*z*	*U*_iso_*/*U*_eq_	
Cu1	0.09934 (3)	0.23847 (2)	0.104081 (19)	0.03448 (11)	
O1	0.20916 (16)	0.08774 (13)	0.10007 (12)	0.0391 (3)	
O2	0.39441 (16)	−0.09065 (15)	0.07532 (13)	0.0489 (4)	
O3	−0.02563 (16)	0.38826 (14)	0.12039 (12)	0.0417 (4)	
O4	0.25659 (17)	0.34556 (16)	0.06197 (12)	0.0464 (4)	
O5	0.40681 (19)	0.48337 (19)	0.13003 (19)	0.0784 (7)	
O6	0.2791 (2)	0.3648 (2)	0.21931 (14)	0.0662 (5)	
O7	−0.45344 (19)	0.13264 (18)	0.18452 (13)	0.0589 (5)	
H1O	−0.5371	0.1148	0.1455	0.071*	
H2O	−0.4318	0.0772	0.2358	0.071*	
N1	−0.07247 (18)	0.14998 (17)	0.13266 (12)	0.0321 (4)	
N2	−0.1848 (2)	0.23436 (17)	0.14647 (14)	0.0362 (4)	
H2N	−0.2694	0.2092	0.1591	0.043*	
N3	0.31622 (19)	0.39954 (18)	0.13960 (18)	0.0477 (5)	
C1	0.1588 (2)	−0.02931 (19)	0.10347 (14)	0.0323 (4)	
C2	0.0145 (2)	−0.0638 (2)	0.11923 (14)	0.0336 (4)	
C3	−0.0258 (3)	−0.1947 (2)	0.11989 (16)	0.0404 (5)	
H3	−0.1210	−0.2163	0.1288	0.048*	
C4	0.0720 (3)	−0.2894 (2)	0.10779 (16)	0.0435 (5)	
H4	0.0438	−0.3747	0.1087	0.052*	
C5	0.2160 (3)	−0.2570 (2)	0.09391 (17)	0.0405 (5)	
H5	0.2833	−0.3214	0.0865	0.049*	
C6	0.2581 (2)	−0.1313 (2)	0.09123 (15)	0.0351 (4)	
C7	−0.0955 (2)	0.0287 (2)	0.13384 (15)	0.0360 (5)	
H7	−0.1876	−0.0002	0.1447	0.043*	
C8	0.5026 (3)	−0.1853 (3)	0.0650 (2)	0.0549 (6)	
H8A	0.5203	−0.2318	0.1251	0.082*	
H8B	0.5904	−0.1446	0.0494	0.082*	
H8C	0.4699	−0.2432	0.0134	0.082*	
C9	−0.1505 (2)	0.3575 (2)	0.13847 (15)	0.0371 (5)	
C10	−0.2645 (3)	0.4550 (2)	0.14953 (19)	0.0504 (6)	
H10A	−0.2274	0.5200	0.1944	0.076*	
H10B	−0.3470	0.4149	0.1742	0.076*	
H10C	−0.2929	0.4931	0.0871	0.076*	

### Atomic displacement parameters (Å^2^)

**Table:**

	*U*^11^	*U*^22^	*U*^33^	*U*^12^	*U*^13^	*U*^23^
Cu1	0.03077 (17)	0.02852 (15)	0.04481 (18)	−0.00120 (9)	0.00701 (11)	−0.00130 (10)
O1	0.0317 (8)	0.0291 (7)	0.0572 (9)	−0.0018 (6)	0.0082 (7)	−0.0017 (6)
O2	0.0358 (9)	0.0386 (9)	0.0730 (11)	0.0047 (7)	0.0088 (8)	−0.0013 (8)
O3	0.0378 (8)	0.0344 (8)	0.0534 (9)	0.0008 (6)	0.0068 (7)	−0.0025 (7)
O4	0.0435 (9)	0.0413 (9)	0.0556 (10)	−0.0083 (7)	0.0114 (7)	−0.0026 (7)
O5	0.0368 (10)	0.0432 (11)	0.154 (2)	−0.0105 (8)	0.0007 (12)	−0.0053 (12)
O6	0.0606 (12)	0.0783 (14)	0.0591 (12)	0.0034 (10)	0.0021 (10)	−0.0132 (10)
O7	0.0467 (10)	0.0621 (12)	0.0672 (12)	−0.0145 (8)	0.0010 (9)	0.0066 (9)
N1	0.0288 (8)	0.0329 (9)	0.0349 (9)	0.0014 (7)	0.0042 (7)	−0.0006 (7)
N2	0.0283 (9)	0.0406 (10)	0.0404 (10)	0.0032 (7)	0.0065 (8)	−0.0008 (7)
N3	0.0280 (9)	0.0326 (10)	0.0819 (16)	0.0059 (8)	0.0016 (10)	−0.0071 (10)
C1	0.0359 (11)	0.0303 (10)	0.0300 (10)	−0.0017 (8)	−0.0009 (8)	0.0011 (8)
C2	0.0366 (11)	0.0336 (10)	0.0304 (10)	−0.0023 (8)	0.0030 (8)	0.0009 (8)
C3	0.0422 (13)	0.0352 (12)	0.0439 (12)	−0.0087 (9)	0.0051 (10)	0.0011 (10)
C4	0.0575 (15)	0.0283 (10)	0.0442 (12)	−0.0059 (10)	0.0011 (11)	0.0021 (9)
C5	0.0499 (14)	0.0320 (11)	0.0386 (12)	0.0059 (9)	−0.0008 (10)	0.0000 (8)
C6	0.0358 (11)	0.0354 (11)	0.0335 (10)	0.0018 (9)	0.0011 (8)	−0.0005 (8)
C7	0.0315 (11)	0.0403 (12)	0.0364 (11)	−0.0064 (8)	0.0033 (9)	0.0012 (9)
C8	0.0411 (13)	0.0506 (15)	0.0730 (17)	0.0135 (11)	0.0048 (12)	−0.0068 (13)
C9	0.0374 (12)	0.0396 (11)	0.0337 (11)	0.0045 (9)	−0.0007 (8)	−0.0009 (9)
C10	0.0473 (14)	0.0481 (14)	0.0555 (14)	0.0140 (11)	0.0023 (11)	0.0005 (11)

### Geometric parameters (Å, °)

**Table:**

Cu1—N1	1.9134 (18)	C1—C2	1.422 (3)
Cu1—O1	1.8798 (15)	C1—C6	1.428 (3)
Cu1—O3	1.9730 (16)	C2—C3	1.419 (3)
Cu1—O4	1.9663 (16)	C2—C7	1.433 (3)
Cu1—O6	2.559 (2)	C3—C4	1.363 (4)
O1—C1	1.312 (2)	C3—H3	0.9300
O2—C6	1.370 (3)	C4—C5	1.407 (3)
O2—C8	1.425 (3)	C4—H4	0.9300
O3—C9	1.248 (3)	C5—C6	1.373 (3)
O4—N3	1.286 (3)	C5—H5	0.9300
O5—N3	1.229 (3)	C7—H7	0.9300
O6—N3	1.231 (3)	C8—H8A	0.9600
O7—H1O	0.92	C8—H8B	0.9600
O7—H2O	0.92	C8—H8C	0.9600
N1—C7	1.286 (3)	C9—C10	1.486 (3)
N1—N2	1.391 (2)	C10—H10A	0.9600
N2—C9	1.333 (3)	C10—H10B	0.9600
N2—H2N	0.8600	C10—H10C	0.9600
			
O1—Cu1—N1	93.67 (7)	C4—C3—C2	121.4 (2)
O1—Cu1—O4	92.90 (7)	C4—C3—H3	119.3
N1—Cu1—O4	171.47 (7)	C2—C3—H3	119.3
O1—Cu1—O3	173.76 (6)	C3—C4—C5	119.5 (2)
N1—Cu1—O3	81.49 (7)	C3—C4—H4	120.3
O4—Cu1—O3	92.30 (7)	C5—C4—H4	120.3
O1—Cu1—O6	97.25 (7)	C6—C5—C4	120.7 (2)
O3—Cu1—O6	82.93 (7)	C6—C5—H5	119.7
O4—Cu1—O6	55.16 (7)	C4—C5—H5	119.7
N1—Cu1—O6	129.12 (7)	O2—C6—C5	124.8 (2)
C1—O1—Cu1	125.84 (13)	O2—C6—C1	113.64 (18)
C6—O2—C8	117.93 (18)	C5—C6—C1	121.6 (2)
C9—O3—Cu1	112.53 (14)	N1—C7—C2	122.85 (19)
N3—O4—Cu1	106.34 (13)	N1—C7—H7	118.6
H1O—O7—H2O	115.6	C2—C7—H7	118.6
C7—N1—N2	119.75 (18)	O2—C8—H8A	109.5
C7—N1—Cu1	128.43 (15)	O2—C8—H8B	109.5
N2—N1—Cu1	111.66 (13)	H8A—C8—H8B	109.5
C9—N2—N1	114.45 (17)	O2—C8—H8C	109.5
C9—N2—H2N	122.8	H8A—C8—H8C	109.5
N1—N2—H2N	122.8	H8B—C8—H8C	109.5
O5—N3—O6	123.6 (2)	O3—C9—N2	119.85 (19)
O5—N3—O4	118.1 (2)	O3—C9—C10	121.7 (2)
O6—N3—O4	118.24 (19)	N2—C9—C10	118.4 (2)
O1—C1—C2	125.80 (19)	C9—C10—H10A	109.5
O1—C1—C6	117.19 (18)	C9—C10—H10B	109.5
C2—C1—C6	117.01 (18)	H10A—C10—H10B	109.5
C3—C2—C1	119.86 (19)	C9—C10—H10C	109.5
C3—C2—C7	117.3 (2)	H10A—C10—H10C	109.5
C1—C2—C7	122.82 (19)	H10B—C10—H10C	109.5
			
N1—Cu1—O1—C1	7.99 (17)	C1—C2—C3—C4	1.5 (3)
O4—Cu1—O1—C1	−166.57 (17)	C7—C2—C3—C4	−179.2 (2)
N1—Cu1—O3—C9	−1.29 (15)	C2—C3—C4—C5	−0.3 (3)
O4—Cu1—O3—C9	172.83 (15)	C3—C4—C5—C6	−0.8 (4)
O1—Cu1—O4—N3	−101.08 (13)	C8—O2—C6—C5	−2.9 (3)
O3—Cu1—O4—N3	75.48 (14)	C8—O2—C6—C1	178.26 (19)
O1—Cu1—N1—C7	−7.63 (19)	C4—C5—C6—O2	−178.0 (2)
O3—Cu1—N1—C7	176.33 (18)	C4—C5—C6—C1	0.7 (3)
O1—Cu1—N1—N2	177.15 (13)	O1—C1—C6—O2	−1.3 (3)
O3—Cu1—N1—N2	1.11 (13)	C2—C1—C6—O2	179.32 (18)
C7—N1—N2—C9	−176.51 (19)	O1—C1—C6—C5	179.8 (2)
Cu1—N1—N2—C9	−0.8 (2)	C2—C1—C6—C5	0.4 (3)
Cu1—O4—N3—O5	−171.45 (16)	N2—N1—C7—C2	179.38 (18)
Cu1—O4—N3—O6	8.3 (2)	Cu1—N1—C7—C2	4.5 (3)
Cu1—O1—C1—C2	−5.8 (3)	C3—C2—C7—N1	−178.50 (19)
Cu1—O1—C1—C6	174.91 (14)	C1—C2—C7—N1	0.8 (3)
O1—C1—C2—C3	179.18 (19)	Cu1—O3—C9—N2	1.2 (3)
C6—C1—C2—C3	−1.5 (3)	Cu1—O3—C9—C10	−177.50 (16)
O1—C1—C2—C7	−0.1 (3)	N1—N2—C9—O3	−0.3 (3)
C6—C1—C2—C7	179.19 (18)	N1—N2—C9—C10	178.48 (18)

### Hydrogen-bond geometry (Å, °)

**Table:**

*D*—H···*A*	*D*—H	H···*A*	*D*···*A*	*D*—H···*A*
N2—H2N···O7	0.86	1.95	2.801 (3)	174
O7—H1O···O1^i^	0.92	2.40	3.271 (3)	159
O7—H1O···O2^i^	0.92	2.42	3.050 (3)	126
O7—H2O···O5^ii^	0.92	2.08	2.984 (3)	167

Symmetry codes: (i) *x*−1, *y*, *z*; (ii) −*x*, *y*−1/2, −*z*+1/2.
